# Darwin’s Other Dilemmas and the Theoretical Roots of Emotional Connection

**DOI:** 10.3389/fpsyg.2019.00683

**Published:** 2019-04-12

**Authors:** Robert J. Ludwig, Martha G. Welch

**Affiliations:** ^1^Department of Pediatrics, Columbia University Irving Medical Center, New York, NY, United States; ^2^Department of Psychiatry, Columbia University Irving Medical Center, New York, NY, United States; ^3^Department of Anatomy and Cell Biology, Columbia University Irving Medical Center, New York, NY, United States

**Keywords:** vagal tone, attachment theory, instinct, calming cycle theory, autonomic conditioning

## Abstract

Modern scientific theories of emotional behavior, almost without exception, trace their origin to Charles Darwin, and his publications *On the Origin of Species* (1859) and *The Expression of the Emotions in Man and Animals* (1872). The most famous dilemma Darwin acknowledged as a challenge to his theory of evolution through natural selection was the incomplete Sub-Cambrian fossil record. However, Darwin struggled with two other rarely referenced theoretical and scientific dilemmas that confounded his theories about emotional behavior. These included (1) the origin of social instincts (e.g., altruism, empathy, reciprocity and cooperation) and the reasons for their conservation in evolution and (2) the peripheral control of heart rate vis-à-vis emotional behavior outside of consciousness. Darwin acknowledged that social instincts are critical to the survival of some species, but had difficulty aligning them with his theory of natural selection in humans. Darwin eventually proposed that heart rate and emotions are controlled via one’s intellect and cortical mechanisms, and that instinctive behavior is genetically programmed and inherited. Despite ongoing efforts, these two theoretical dilemmas are debated to this day. Simple testable hypotheses have yet to emerge for the biological mechanisms underlying instinctive behavior or the way heart rate is controlled in infants. In this paper, we review attempts to resolve these issues over the past 160 years. We posit that research and theories that supported Darwin’s individualistic brain-centric and genetic model have become an “orthodox” Western view of emotional behavior, one that produced the prevailing behavioral construct of attachment as developed by John Bowlby. We trace research and theories that challenged this orthodoxy at various times, and show how these challenges were repeatedly overlooked, rejected, or misinterpreted. We review two new testable theories, *emotional connection theory* and *calming cycle theory*, which we argue resolve the two dilemmas We show emerging scientific evidence from physiology and a wide variety of other fields, as well from clinical trials among prematurely born infants, that supports the two theories. Clinical implications of the new theories and possible new ways to assess risk and intervene in emotional, behavioral and developmental disorders are discussed.

## Introduction

The modern scientific study of emotions began with Darwin’s comparative studies of animals and humans and soon inspired the fields of psychology and physiology, among many others. To this day, however, Darwin’s assumptions about emotional behavior persist as a kind of “orthodoxy” throughout science and society, one that has been remarkably resistant to change or challenge over the ensuing 160 years.

Great breakthroughs and advances have been made in science and medicine since Darwin. We can coax a body’s own immune system to attack a tumor, eradicate viral and bacterial infection, fix heart defects, identify and correct for genetic disorders, and overcome the most debilitating injuries. We can also extend the life span and save infants born at twenty-two weeks of gestation. However, when it comes to understanding and treating emotional and behavioral disorders that arise in infancy and early childhood, science has made relatively little progress since Darwin (Dong et al., 2012; Lean et al., 2017). Despite exponential increases in research over the last 40 years, there is little evidence that science is any closer to fully understanding autism spectrum disorder (ASD), let alone curing it (French and Kennedy, 2018). The same can be said about Attention Deficit Hyperactivity Disorder (ADHD) (Catala-Lopez et al., 2017), school-age child developmental disorders (Kingston and Tough, 2014), post-traumatic stress disorder (de Graaff et al., 2018), and a host of other psychiatric and neurodegenerative disorders. Where signs of success in the treatment and prevention of emotional and behavioral disorders have appeared (Moore et al., 2016; Moreno-Peral et al., 2017), the lack of scientific theories that can explain their efficacy have hampered efforts to change standard medical care. All in all, emotional and behavioral disorders in infants and young children continue to be daunting challenges to science (Cameron et al., 2017), and among the greatest burdens to modern society (Younger, 2016).

It is indeed perplexing that our relative lack of progress has failed to prompt a fundamental rethinking of the way we view human emotions and behavior. In most scientific fields, longstanding failure to explain anomalies and solve challenges typically prompts a rethinking of the theoretical underpinnings of the discipline (Frontiers Collection, 2015). Where would we be, for instance, if 100 years ago Einstein had not challenged conventional assumptions in order to explain dilemmas that Newtonian physics could not explain? We suggest that it is time to question the underlying assumptions and beliefs about human emotion as a means to finding new ways to treat emotional and behavioral disorders.

In this review, we historically track efforts to resolve two rarely referenced dilemmas that challenged Darwin’s assumptions about emotional behavior. These dilemmas are separate from Darwin’s well-known, self-acknowledged inability to explain the incomplete fossil record below the Cambrian explosion at the time (Conway, 2006). One dilemma for Darwin was that he could not explain instinctive (innate) behaviors that emerge in the perinatal period of development. What could account for the behaviors of a mother and baby soon after birth, especially the instincts of empathy and altruism? Still another dilemma was that he could not explain peripheral control of heart rate. Darwin pondered if, as his theory posited, emotional behavior is controlled via the central nervous system, how can heart rate be inhibited separately by peripheral influences? What is the mechanism and function of peripheral inhibition of heart rate? Darwin believed that these two dilemmas needed to be answered in order to fully explain human emotions.

The two dilemmas raised heated political arguments about the origin and nature of emotions that took on ideological dimensions. To “compete and dominate” vs. to “empathize and care” foreshadowed the great 20th century ideological and political split, pitting Capitalism, with *Natural Selection* as its raison d’etre (Spencer, 1984), against Communism, which made *Cooperation and Mutual Aid* its sources of inspiration (Adams, 2016). The problem of how heart rate is controlled was equally controversial, with psychologists favoring cortical control pitted against physiologists presenting data that challenged that view.

Darwin considered the problems of instinctive behavior and heart rate control to be connected. Through discussing them in the new evolutionary light of natural selection, he questioned why heart rate and emotions, positive and negative, can be influenced both by conscious will and unconscious physiological mechanisms. The idea that man’s emotions *might not* be controlled by higher order consciousness – his God-given superior human intellect and will – *the very things that were believed to separate him from inferior species* – was blasphemous and unthinkable to religious Victorian England. Darwin did his best to walk the line between what he believed, what his data showed, and what his audience did not want to hear. And, he left it to his followers to sort out the reality.

While this dichotomy had a profound effect upon political debate, we limit our review to the theoretical scientific story, which we follow separately and chronologically for each dilemma. In Part 1, we present Darwin’s thoughts on instincts, along with competing scientific evidence that emerged from the study of instincts over the ensuing 160 years. In Part 2, we follow Darwin’s discussions on peripheral control of heart rate and review the various competing theories and evidence that emerged through the present. In Part 3, we first critically review John Bowlby’s prevailing behavioral construct of attachment and discuss why the construct is not useful in fully understanding, assessing and treating emotional behavior. Following this, we critically review evolutionary game theory and discuss why it does not fully address fundamental problems identified by Darwin. We explain why polyvagal theory provides a partial explanation for the problem of peripheral control of rate, but does not provide an adequate mechanism or theory of change. We also review the authors’ calming cycle theory, which provides a novel explanation for how socioemotional behavior is governed in an interpersonal co-regulatory manner via bottom-up sub-cortical *Pavlovian conditioning* and *visceral/autonomic learning* mechanisms, along with evidence that supports this new theory. Finally, we review the authors’ theoretical construct of *emotional connection* and explain why it is different from attachment, why it has more predictive value and is more useful in assessing and treating behaviors of infants and mothers, and the data from recent clinical trials that supports the construct.

## Part 1: Darwin’s Social Instinct Dilemma

### Overview

Throughout his writings, Darwin searched for a rationale to explain the evolution of “social instincts” in light of his *Principle of Natural Selection* (Darwin, 1859). He argued that humans with superior intellect, who exhibit competitive characteristics attuned to and advantageous for their specific environment, will most likely survive, reproduce, and pass on their genes. Because of this, subsequent generations are more likely to possess those advantageous characteristics. Herbert Spencer popularized this idea with the phrase “survival of the fittest,” partly based on the Lamarckian belief that struggle for survival led to traits that could be inherited.

In his first book, Darwin used the term instinct or innate behavior 152 times and acknowledged that instincts presented a challenge to his theory. He asked, “Can *instincts* be acquired and modified through natural selection?” (Darwin, 1859). Without offering a definition for instinct, he acknowledged:

*An action, which we ourselves should require experience to enable us to perform, when performed by an animal, more especially by a very young one, without any experience, and when performed by many individuals in the same way, without their knowing for what purpose it is performed, is usually said to be instinctive* (Darwin, 1859).

An action repeated over and over can become a habit that is performed without thinking, but Darwin argued that such action (habit) is different from an instinct. Citing multiple examples from the animal world, Darwin concluded:

“ … *metaphysicians have compared instinct with habit. This comparison gives, I think, a remarkably accurate notion of the frame of mind under which an instinctive action is performed, but not of its origin*” (Darwin, 1859).

A decade later and with evolved thinking, Darwin distanced himself from Spencer’s “*survival of the fittest*” sentiments in *The Descent of Man* (Darwin, 1871) by acknowledging that widely exhibited traits like cooperation, empathy, reciprocity and altruism are sometimes crucial to a species’ ability to survive. Such traits, he mused, could be an after-effect of a group of behaviors underlying instincts. “The aid which we feel impelled to give to the helpless,” he wrote, “is mainly an incidental result of *the instinct of sympathy*, which was originally acquired as part of *the social instincts*, but [this instinct was] subsequently rendered … more tender and more widely diffused” (Darwin, 1871).

Nonetheless, this reasoning did not resolve the dilemma for Darwin. He wrote, “It is extremely doubtful whether the offspring of the more sympathetic and benevolent parents, or of those which were the most faithful to their comrades, would be reared in greater number than the children of selfish and treacherous parents of the same tribe” (Darwin, 1871). Darwin argued that genes of cooperative altruistic humans, if passed on, would ultimately weaken and degrade the species. To support his argument, he pointed to the practice of animal husbandry, which had over thousands of years improved the quality of livestock by culling out inferior and weak individuals, without regard to their suffering. Only humans, he complained, protect and nurture their weak and infirm.

Darwin wrote extensively about emotional expression in both humans and animals. He incorporated contemporary insights from natural observations to frame the problem of instincts within his first two principles of emotional expression and behavioral habits (Darwin, 1872). His description of emotional instinct was closely tied to observations in the postnatal period and was partially inspired by the behavior of his own first-born child. He concluded that behaviors could not be the result of habits, noting, “The far greater number of the movements of expression, and all the more important ones, are, as we have seen, innate or inherited; *and such cannot be said to depend on the will* [i.e., cognition] of the individual” (Darwin, 1872).

Darwin summarized all emotional expressions in animals and humans with three general principles [reviewed in-depth by Hess and Thibault (2009)]. First, *the principle of serviceable habits* stated that certain useful habitual emotional expressions or “instincts” are acquired over time through experience by the species and are genetically inherited by offspring [a view previously developed by Lamarck (1809)]. Darwin insisted, however, that emotional behaviors had evolved, as many emotional behaviors that were functional in the past or characteristic of lower animals were no longer present in humans. Second, *the principle of antithesis* asserted that some emotional expressions are simply the opposite of serviceable ones and that these become traits, in addition to physiological and psychological states. Over time, certain emotional expressions became habitualized or ritualized in order to act out an emotion, as occurs in courtship or fighting. Third, *the principle of the direct action of the excited nervous system on the body* stated that some emotional expressions are universal and are directly associated with an underlying emotional state. For example, Darwin described laughter as quasi-convulsive movement that discharges an overflow of nervous energy induced by either physical or psychological tension.

**FIGURE 1:**
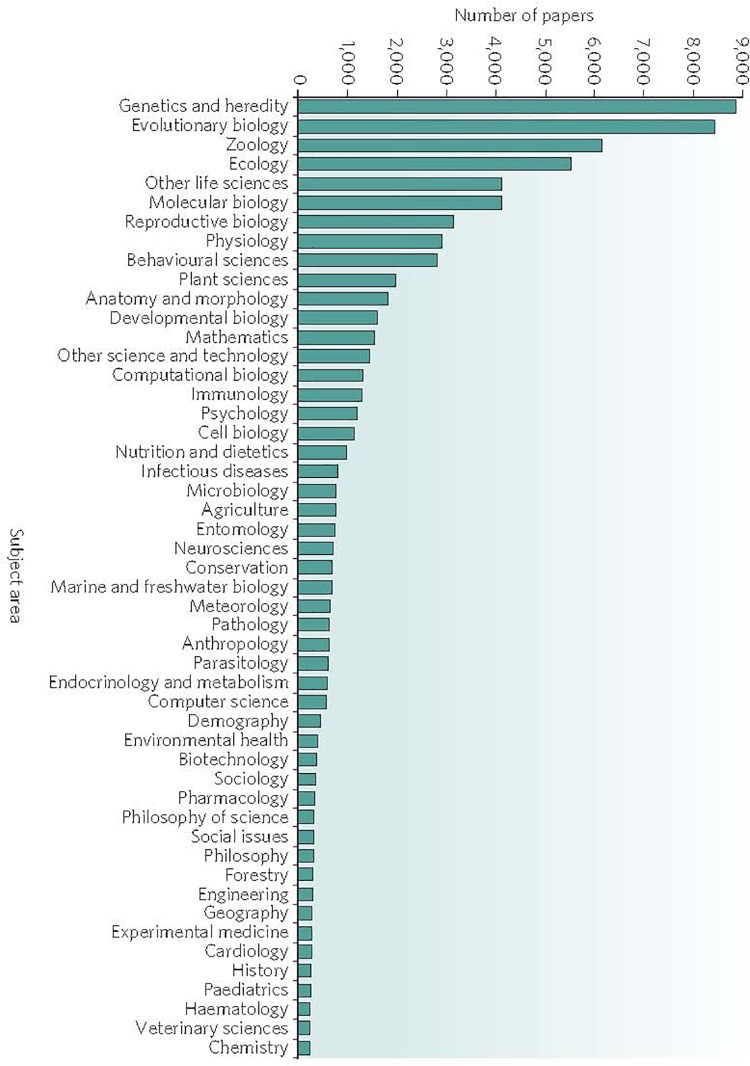
Search by subject area completed in November 2008 by Mark Pagel on the ISI Web of Science data base as showing partial list (∼40,000) of scientific papers that include the term “natural selection” in their title, abstract or key words. Reprinted with permission from Springer Nature (Pagel, 2009). Note that the subject area “Genetics and Heredity” generated the largest number of published papers. Epigenetics does not appear on the list. By contrast, a 2019 search for the words “natural selection” and “genetics” on PubMed returned over 7,000 papers, whereas a search for “natural selection” and “epigenetics” returned only 65 papers.

These three principles laid a groundwork for the scientific investigation into emotions and emotional behavior that influenced multiple new scientific fields; genetics, electrophysiology, cognitive neuroscience, neurology, psychology, ethology, endocrinology and computer science, to name a few (Figure 1) (Pagel, 2009). Darwin’s insights and theories permitted researchers to make new inferences about human behaviors, such as learning mechanisms, memory, emotions, and social interactions, based on animal and human observations and experiments. In this respect, Darwin’s collective writings, rather than resolving the dilemma of instincts, have served as source documents that have encouraged ongoing scientific investigations into emotions and instincts.

### Attempts to Resolve the Social Instinct Dilemma

Prior to Darwin, instinctive behavior was largely explained through the nativism teachings of Plato and Descartes, which assumed that a God or a similar being, or a process, placed innate ideas and principles in the human mind. In the decades following Darwin, such behavior was being studied by a rapidly growing number of entomologists and ethologists who were cataloging instinctive behaviors.

Entomologist Jean Henri Fabre (1823–1915) spent his life observing the behavior of insects. He considered instinct to be any behavior that did not require cognition or consciousness to perform. The American amateur zoologist and ethologist Charles O. Whitman (1842–1910) revolutionized thinking about the physiological mechanisms by which hormones influence behavior and launched the field of behavioral endocrinology (Marler, 2005). But it was C. Lloyd Morgan (1852–1936), more than any other theorist, who tried to resolve the confusion over natural selection and inherited habit (Morgan, 1896).

Morgan drew heavily on the experimental work of physiologist Charles S. Sherrington (1857–1952), who demonstrated that complex reflex responses could still be elicited in animals with their cerebrum removed. Morgan argued strenuously that instincts at birth are subconscious reflexes that lay in the inherited arrangement of neural connections found in the *lower brain centers* (Morgan, 1912). However, under intense resistance from researchers who were focused on reflexes that require higher-order consciousness, Morgan agreed to a definition of reflex that included both subconscious *and* conscious actions. Thus, Morgan’s ultimate explanation, which formed the theoretical foundation of Neo-Darwinism in the twentieth century, left the definition of reflex broad enough to be confusing and therefore Darwin’s dilemma of instincts unresolved (Richards, 1977).

Two other researchers who focused on conscious behaviors were German physician and physiologist Wilhelm Maximilian Wundt (1832–1920) and psychologist Edward Titchener (1867–1927), who helped found a new independent field of *Experimental Psychology*. Together, they developed the concept of *Structuralism* to analyze the adult mind as the total sum of experience from birth to the present. Titchener’s method for analysis focused on introspection via self-reports of sensations, views, feelings and emotions to establish the simplest definable emotional components of one’s life, so that they may be reconstructed and more fully understood (Gozli and Deng, 2018).

Among the researchers invigorated by Darwin’s ideas about natural selection and cognition was the German neurologist Ernst Haeckel (1834–1919), whose “biogenetic” principle played a major role in the thinking of Sigmund Freud (1856–1939), both in the formation of psychoanalytic theory in general, and its metapsychology in particular (Makari, 2008).

Freud incorporated aspects of Darwin’s ideas about survival and emotional behavior into his concepts of psychoanalysis. For instance, Darwin’s first principle of the expression of emotions, especially his definition of instinct as “inherited learning,” had a clear continuation in Freud’s psychoanalytic theory. It posits that a specific state of mind is associated with a habit or movement, and can be repressed by will. This repression of instinctive or innate feelings, mind over body, is a hallmark of psychoanalytic theory and psychopathology.

Freud accepted that natural selection is genetic in nature, yet postulated that instinct and emotion can be understood by examining the mind. Thus, Freud failed to address the philosophical dilemmas that natural selection posed for Darwin. Freud adopted much of Haeckel’s theory and the ideas and methods of Titchener, which emphasized introspection, self-reflections and hypnosis as the gateways to understanding emotional behavior (Ritvo, 1974).

At the turn of the twentieth century the argument was largely dominated by Neo-Darwinists, who accepted Darwin’s evolutionary theory of natural selection without resolving the theoretical dilemmas it posed. Ethologists, meanwhile, were busy cataloging the developmental aspects of perinatal parent/infant instinctive emotional behaviors in animals and insects. Aspects of behavior in humans were being explored by psychologists, who were split between those who were influenced by the psychodynamic approach of Freud and those following a new school of *Behaviorism*. Both groups were increasingly interested in abnormal emotional behaviors that deviated from normal instinctive behaviors soon after birth and the relationship between these early abnormal behaviors and emotional problems later in life.

Meanwhile, Freud’s proposed genetic basis for instinctive behavior was generally accepted in the field of psychology as the key to understanding neuroses. The scientific climate in the late 1800’s formed a strong base for Freud’s theoretical thinking and for the growth of psychoanalysis, but advances in biology were beginning to challenge the basis for this synthesis at the beginning of the twentieth century (Hofer, 2014). Calls for scientifically testable hypotheses in psychology began to grow. Even Freud recognized the problem, writing in 1925, “There is no more urgent need in psychology than for a securely founded theory of the instincts” (Freud, 1925).

This search was already well underway. V. M. Bekhterev (1857–1927) was the first to employ the use of experimental methods to study the central nervous system in psychology. His research on associated responses became highly influential in a new branch of psychology called *behaviorism* (later the basis of *Reflexology*, *Gestalt Psychology*). John B. Watson (1878–1958), an early proponent of behaviorism, attempted to make psychology scientifically relevant and acceptable, adding his voice to a growing chorus of basic researchers criticizing Freud’s nebulous and metaphoric ideas about “consciousness.”

“*The position is taken here that the behavior of man and the behavior of animals must be considered on the same plane; as being equally essential to a general understanding of behavior. It can dispense with consciousness in a psychological sense…the findings of psychology become the functional correlates of structure and lend themselves to explanation in physico-chemical terms*” (Watson, 1913).

Parting completely from Darwin and Freud, Watson proposed that *nothing is instinctual in the infant*. Rather, Watson stated that everything is built into a child through the interaction with his environment. Impressed with Pavlov’s early work on learning, he incorporated a highly simplified version of Pavlov’s principles of the conditional reflex and autonomic conditioning into his behaviorism concepts and experiments.

By the 1940s, however, two other American behaviorists, B. F. Skinner (1904–1990) and Edward Lee Thorndike (1874–1949), were modifying the ideas of Watson and embracing Konorski’s cognitive learning mechanism. Skinner believed that Pavlovian conditioning reflex was too simplistic to explain complex human behavior. He looked for the causes of an action and its consequences, calling his cognitive learning approach *operant conditioning* (i.e., changing behavior by following a desired response with reinforcement) (Skinner, 1988). This method followed Thorndike’s *Law of Effect*, which states behavior that is reinforced tends to be repeated and, that behavior not reinforced tends to be extinguished.

Despite the growing emphasis on a cognitive and mechanistic view of human behavior among psychologists, ethologists continued to study instinctive behavior in the animal world between 1900 and 1950. American experimental psychologist and behavior scientist Wallace Craig (1876–1954) studied the way innate and learned emotional behavioral tendencies are integrated with evolutionary, motivational, experiential, social and ecological degrees of freedom, and how vocal and social behaviors are organized (Craig, 1922). The work of Dutch biologist Nikolaas Tinbergen (1907–1988) (Tinbergen, 1951) and Austrian biologists Konrad Lorenz (1903–1989) and Karl von Frisch (1888–1982) combined laboratory and field science with other disciplines, such as neuroanatomy, ecology, and evolutionary biology, to study aggression, “nurture” and “bonding” in animals. Tinbergen did not venture into theoretical matters, but instead focused his efforts on standardizing the study of instincts in the field. His methods continue to be the gold standard for fully explaining behavior (Bateson and Laland, 2013).

Lorenz, on the other hand, theorized prolifically. He gained acclaim and popularity for his theory on the evolution of instinctive animal communication, based on his observations of fixed action patterns of geese and their ability to bond to humans after birth. Lorenz popularized the notion of *imprinting*, whereby a newly hatched chick forms what he termed an “attachment” to the person caring for it and follows it around. From his observations, Lorenz concluded that the attachment phenomenon provided compelling evidence that social instincts are not learned through experience. Rather, he concluded disturbingly, they are inherited, genetic and *decadent* (Kalikow, 1983).

The research of ethologist Lorenz and the beliefs of Freudian psychologists produced a similar consensus that instincts are inherited, genetic in origin, and more or less fixed at birth. These theories and ideas collectively formed the foundational principles of the new interdisciplinary field of *neuroscience*, which was bringing together the fields of Physiology, Behavioral Psychology and Genetics, among others. A general excitement abounded at the mid-point of the twentieth century that Darwin’s dilemmas could finally be explained and resolved through increasingly reductive brain and gene research.

It was at this time and in this theoretical climate that British psychiatrist and researcher, John Bowlby (1907–1990) set out to find a testable hypothesis to Freud’s ideas on instincts. He did so by compiling theories and research that prevailed at the time. Mary Ainsworth, Bowlby’s protégé, explained his goal in 1969:

“[Bowlby] *proposes to replace Freudian instinct theory with a set of propositions, testable through research, more closely in line with present-day knowledge, while at the same time respecting the many psychoanalytic contributions to understanding human experience and behavior which are not tied inextricably to an antiquated instinct model*” (Ainsworth, 1969).

It is difficult to follow Bowlby’s theory and the arguments supporting it, mainly because the theory is not a tightly articulated or consistent theory but a wide-ranging and sometimes contradictory amalgamation of theories from various fields. Nonetheless, in Part 2 of his first book (Bowlby, 1969), Bowlby claims that he has created a new comprehensive explanation for instinctive behavior:

“*A long-awaited theoretical breakthrough has been achieved by analytical biology and control theory, which together have elucidated the basic principles that underlie adaptive, goal-directed behavior. Exploiting this breakthrough have been three empirically based sciences: ethology, experimental psychology, and neurophysiology*.”

Bowlby stated that his new *Attachment Theory* was built upon the Neo-Darwinist genetic theory of natural selection and Freud’s drive theory. Citing Williams (1972), Weiner (1948), Skinner (1938), Lorenz (1937), Hinde (1970) and various cognitive scientists and their research, Bowlby proposed that behavioral systems are individual and self-controlled through feedback loops in a hierarchical top-down manner in ways that promote and satisfy the individual’s needs and survival instincts. Thus, Bowlby places his work solidly within the Darwinian orthodox theoretical mainstream without offering any new insights on the two dilemmas discussed here.

Bowlby did part slightly from Freud on instinctive behavior, postulating that rather than inheriting the instinct itself one inherits a “potential” for the instinct. In the end, however, Bowlby, like Darwin before him, did not offer a definition of instinctive behavior, but rather offered a description of it. Bowlby’s ideas about early infancy and early childhood, as well as the research they stimulated, were soon criticized as lacking rigorous scientific validation (Mercer, 2011) or theoretical rigor (Lehrman, 1953). Despite these shortcomings, however, Bowlby’s description of behavior associated with the Attachment construct and Ainsworth’s behavioral coding system (Crittenden, 2017) remain the standards by which psychologists assess and treat behavior.

In the late 1960’s and 1970’s, influenced by a group of child development researchers at Harvard University, Colwyn Trevarthen (born 1931) developed the theory of “innate intersubjectivity” (Trevarthen and Aitken, 2001) to explain the emergence and development of active “self-and-other” awareness in infancy. Intersubjectivity theory proposes that “the infant is born with awareness specifically receptive to subjective states in other persons,” and that a human individual “grows in active engagement with an environment of human factors – *organic at first*, then psychological or inter-mental.” The terms “innate” and “organic” are not clearly defined and no pre-natal learning mechanism is proposed. Thus, intersubjectivity theory focuses on the psychological mechanisms associated with social development following birth, leaving the dilemma of instincts unresolved.

Contemporaneous with, yet completely separate from Bowlby’s work in the 1970’s, American psychiatrist Martha G. Welch reported anecdotal evidence that contradicted accepted theories on instinctive behavior. Welch treated autistic children and their families with an intervention that involved regular and repeated physical “co-calming” sessions, primarily between mothers and children (Welch, 1988). The hallmark of the intervention was that the treatment method led to profound phenotypical changes, from maladaptive avoidant behavior to adaptive approach behavior in both the child and mother, sometimes following a single calming session (Welch et al., 2006).

Welch’s work came to the attention of Nobel Laureate Niko Tinbergen, who had called in his Nobel speech for young clinicians and scientists to take a new look at autism. After visiting Welch’s treatment center and observing phenotypical behavioral changes in autistic children and their mothers, Tinbergen became convinced that Welch had discovered a therapeutic breakthrough. For the last ten years of his life, he and his wife helped promote Welch’s discovery (Welch, 1983). But, by the late 1990’s, it became clear that Welch’s insights were not going to change the treatment of emotional disorders without providing rigorous scientific evidence to support her method, including a biological mechanism and a theory of change to support the behavioral changes observed using her treatment method.

From the late 1940s to the present, various *developmental psychobiologists* sought scientific explanations for ontological questions surrounding prenatal, perinatal and early childhood development and emotional behavior by combining biological psychology, neuroscience and many other areas of biology. Of this group, Myron A. Hofer, was one of the most influential. An early proponent of Bowlby, Hofer came to realize that it was not possible to generate testable hypotheses within Bowlby’s theoretical construct of attachment as a unique motivational system (Hofer, 2006). Instead, Hofer and his colleagues focused their research on the basic biological components underlying mother/infant behaviors, such as sleep, feeding, thermoregulation, attention, and on Michael M. Myers’ work on the specific relationship between behavior and cardiovascular function (Myers, 1992).

Hofer posited that within normal mother–infant interactions there are three main categories of “hidden regulators” associated with the caregiver: behavioral-sensorimotor, thermal-metabolic and nutrient-interoceptive (Hofer, 1994). Using animal models to study specific maternal stimulations, he found that warmth, milk and touch had immediate regulatory effects on various physiological activity, including heart rate (Hofer, 2006). Hofer theorized that these hidden regulators form the biological basis of Bowlby’s “internal working model” of attachment. These regulatory interactions become associated with physical and psychological events (Hofer, 2006), an idea similar to Darwin’s first principle on the expression of emotions (see above). Hofer’s data pointed to autonomic regulation embedded in social interactions between mother and infant, with mother regulating infant and, in turn, infant regulating mother, especially with regard to eliciting maternal behaviors. In experiments involving what he termed “social entrainment of biologic rhythms,” Hofer suggested *Pavlovian learning* could be at work (Hofer, 1984). He postulated that disruption of this mother-infant regulation through social separation can have profound lasting negative impact on various functions of the autonomic nervous system, including heart rate, and that such events can negatively impact physiology throughout the life span.

Yet another group of scientists, *evolutionary biologists* (Payne and Wagner, 2018), were beginning to reveal intriguing new statistical facts about Darwin’s social instincts. Applying new mathematical models in the 1960’s, William Donald Hamilton (1936–2000) proposed a genetic basis for cooperation and altruism that supported Darwin’s theories, based upon the concept of inclusive fitness. Also building on new mathematical breakthroughs, evolutionary game theory originated in 1973, when John Maynard Smith (1920–2004), and George R. Price (1992–1975) proposed mathematical criteria that can be used to analyze and predict contests and competing strategies first identified in animals by ethologists Lorenz and Tinbergen (see above) (Maynard-Smith and Price, 1973). The theory proposed a basis for social instincts in Darwinian evolution by applying game theory to evolving populations in biology (Nowak and Sigmund, 2005).

Today, evolutionary game theory is of interest to economists, anthropologists, and philosophers and to sociobiologists, such as E. O. Wilson (see above). An offshoot of evolutionary game theory, indirect reciprocity theory proposes an explanation of altruistic, cooperative interactions in human populations (Nowak and Sigmund, 2005). This theoretical work has advanced the understanding of certain aspects of human behavior by going beyond the rational, touching on the subconscious and proposing various cognition-based mechanisms that drive emotions, especially with respect to cooperation (Wang et al., 2017; Xia et al., 2017). In that sense, evolutionary game theory ends up supporting Darwin’s theories of natural selection and instincts and falls in line with traditional views on the origin of emotions. While solving certain aspects of social instincts, evolutionary game theory falls short of producing a simple testable hypothesis that can account for epigenetic factors influencing instinctive behavior in the perinatal period. See more on this point in Part 3 below.

About the same time, however, ethologists were accumulating new evidence that suggested evolution might be occurring at the group level, and that social instincts play a key role. Gradually, a new group calling themselves *sociobiologists* (Wilson, 2008b) began to challenge Darwin’s natural selection theory. In 1966, Suzanne Batra (born 1937) introduced the term *eusocial* (meaning “good” social) to describe the nesting behavior of certain species of bees. Batra observed that these bees, males and females alike, displayed social stratification that included cooperative, altruistic behavior during various brood raising tasks within the colony, and that these behaviors gave the group an advantage over competing groups. About the same time, E. O. Wilson (1929) mounted a formidable challenge to the prevailing belief that all behavior was genetic, based on observed altruistic behavior of ants. Wilson theorized that evolution actually tends to favor epigenetic environmental factors and traits, such as empathy, altruism and cooperation, over individual traits. Starting in 1994, E. O. Wilson, together with David Sloan Wilson (born 1949) and Elliot Sober (born 1948), began proposing that *amplification* of social instincts through small mutations in polymorphisms or alleles (*which importantly have to do with how the young are cared for*) has given certain species, including homo sapiens, a phenotypical competitive survival advantage (Sober and Wilson, 1998; Wilson, 2008a).

Due largely to advances in research technology, there was tremendous growth in scientific knowledge about the structure and function of genes during the second half of the twentieth century, thus promising new ways to test genetic theories. Solving the structure of DNA in 1953 by James Watson (1928) and Francis Crick (1916–2004), opened a new era in gene research, and with it predictions that breakthrough treatments of emotional disorders were imminent. This excitement gained even more intensity in the early 1960’s, when the description of nerve signals and synaptic transmission by Julius Axelrod led to a rapid acceleration of biochemical brain research on the effects of psychotropic agents on brain chemistry. Then, with development of DNA sequencing in 1977 by Frederick Sanger (1918–2013), excitement spread to the business world, which quickly monetized these discoveries. By 2000, however, the search for simple genetic explanations and/or brain mechanisms for emotional disorders, such as autism, began to stall as genetic makeup by itself could not account for behavioral variance and anomalies. The stall was driven in large part by a growing body of science that showed epigenetic factors, such as plasticity, played an important role in the etiology and regulation of emotional disorders (Agrawal, 2001; Gardener et al., 2011; Turecki and Meaney, 2016).

Aside from the excitement generated by the new genetic findings, research was revealing ways that genes can be modified behaviorally through experience. In his 1972 essay titled “Ethology,” Tinbergen summarized a large number of studies that demonstrate ways in which innate behavior that is not changed by cognitive learning interacts with environmental experience to produce structured patterns of instinctive behavior (Tinbergen, 1972). Gilbert Gottlieb (1929–2006) showed that transgenerationally recurring prenatal sensory experiences are critical to learning during the prenatal and postnatal periods of development (Blumberg, 2017; Lickliter, 2017). Gottlieb’s theory of *probabilistic epigenesis*, which states that there is no predetermined path to trait development, was the first serious challenge to both the Darwinian and Neo-Darwinian theories of natural selection (Gottlieb, 2002).

The first two decades of the current century have witnessed a sea change in the science of behavior, how the body communicates with and influences cognition and how the brain processes communications from the body to produce behaviors. Neuroscientist Joseph LeDoux popularized the term “Social Brain” (LeDoux, 1996) and identified two integrated sensory pathways to the amygdala, one fast and subcortical and the other slow and cortical (LeDoux and Brown, 2017). Neurobiologist Antonio Damasio mapped the neural systems that underlie emotion, decision-making, memory, language and consciousness. His somatic marker hypothesis (Damasio, 1996) describes how the biological underpinnings of emotions influence both positive and negative decision-making, often subconsciously. Many have worked to merge the latest findings in evolutionary biology as first noted by Darwin, with developmental biology (Kutschera and Niklas, 2004), as well as attachment theory (Lickliter, 2008). This new “evo-devo” field builds on work of SG Mivart (1827–1900), who was one of the first to recognize the limited role of natural selection (Chhetri, 2014). To explain how species evolved from one another, Mivart ceded natural selection as an environmental and efficient cause, but he proposed that instincts within the individual organisms are a formal “cause.”

**FIGURE 2:**
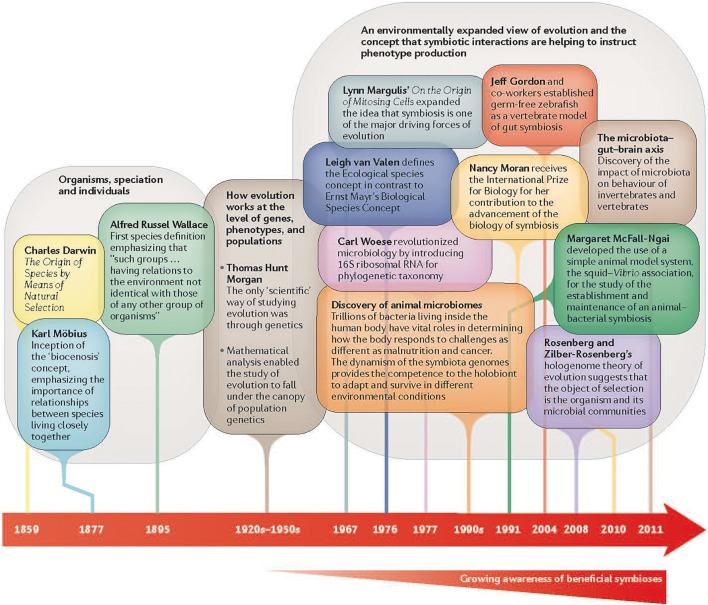
Growing awareness of beneficial symbiosis of organisms to result in an organism’s phenotype. Reprinted with permission from Springer Nature (Gilbert et al., 2015).

**FIGURE 3:**
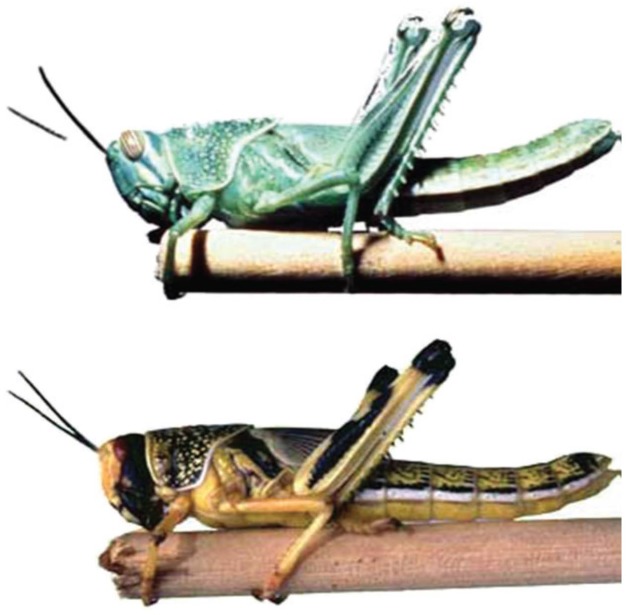
Solitary and gregarious morphs of the desert locust. This dramatic and rapidly produced difference in both appearance and behavior is triggered by increased social stimulation. Reprinted with permission from AAAS (Anstey et al., 2009).

The even newer field of eco-evo-devo adds ecological considerations. This field examines the developmental processes of two organisms in symbiotic relationship to each other to uncover the ways that animal bodies evolved and function (Figure 2). This new field is studying genetic and environmental mechanisms that underlie the development of social and cognitive competencies, as well as the epigenetic (gene–environment interactions) processes that adapt these competencies to local conditions (Lickliter, 2017) (Figure 3).

Another relatively recent scientific breakthrough that has raised appreciation for the power of body functions to shape emotional behavior and cognition is the mapping of the human microbiome. Gut microbiota have been found to have significant effects on cardiac modulation (Polsinelli et al., 2017), mood regulation (Vitetta et al., 2014), immunologic, hormonal and metabolic homeostasis (Lach et al., 2018) and early infant development (de Weerth, 2017). At the same time, gut-brain vagal stimulation was introduced to remediate a wide range of medical, psychiatric and emotional disorders, including autism (Engineer et al., 2017) and G.I. disorders (Bonaz et al., 2018). In animal models of sepsis, the vagus nerve has been proposed to play a crucial role in the regulation of the immune response (Matteoli and Boeckxstaens, 2013). Gut vagal afferents differentially modulate learned fear and innate anxiety, adding further weight to theories emphasizing an important role of afferent visceral signals in the regulation of emotional behavior (Klarer et al., 2014). While microbiome research has shifted attention of science and the public to peripheral influences on behavior and health, for the most part microbiome and vagal signaling findings are still being interpreted within the orthodox theoretical brain-centric self-regulation paradigm that has prevailed since Darwin (Foster et al., 2017).

Since 2000, the ability to examine instincts, or innate behaviors, in humans has grown rapidly, due to several converging factors. Rising premature birth rates coupled with improved survival, now account for one out of ten births in the United States (Ambrose et al., 2015). The maternal separation required by intensive hospitalized care, along with the long-term emotional, behavioral and developmental consequences of these early births (Agrawal et al., 2018), have created opportunities for researchers to test theories about perinatal mother-infant innate behavioral mechanisms in a highly controlled environment. Yet, despite recent growth and diversity in scientific findings, the main medical assumptions that determine how socioemotional behavior is viewed or treated have evolved very little over the past 160 years. Innate behaviors are still regarded as individual and genetic in origin and therefore resistant to change. When symptomatic emotional behavior arises, it is often regarded as psychological in origin and is assessed within Bowlby’s attachment construct and theory (Perry et al., 2017; Mikulincer and Shaver, 2018). The strategies for remediating symptomatic behavior involve various cognitive learning therapies and/or drug therapies targeting the brain. This current thinking and strategy, however, is increasingly criticized as not meeting growing worldwide mental health problems (Patel et al., 2018). Attempts to link Attachment phenotypes to physiology have failed thus far (Barazzone et al., 2018). And, attempts to exploit brain mechanisms using pharmaceuticals and conventional behavioral interventions have proved costly and/or when effective, not scalable (Timlin et al., 2014; Feliu-Soler et al., 2018).

To summarize, there has been a vast amount of research in the past century increasing scientific knowledge about the brain and genes, accompanied by many medical and scientific “reductionist” breakthroughs. Multiple attempts to revise, repackage or reinterpret Darwin’s original conclusions about instincts, based upon the assumptions that he himself was not entirely satisfied with, have yet to produce a simple testable hypothesis when it comes to the emotional behaviors that arise between mother and infant in the perinatal period.

## Part 2: Darwin’s Control of Heart Rate Dilemma

### Overview

While it was known since antiquity that heart rate could be inhibited or even stopped by stimulating the peripheral pneumogastric or vagus nerve without conscious input (Hoff, 1940), the phenomenon was a curiosity to physicians and physiologists. Descartes was among the many prominent scientists who offered theories on peripheral and central inhibition of the heart (Stuart et al., 2014), but the mechanism(s) were not yet understood (Hoff, 1936). This presented a particular problem for Darwin’s theory of natural selection, which favors the behaviors of intellectually superior individuals. If evolution is influenced by conscious mechanisms, what is the role of peripheral and unconscious mechanisms? Darwin was greatly puzzled by the power of the unconscious actions of social instincts, especially in the newborn infant. He stated:

“*Reflex actions*, in the strict sense of the term, are due *to* the excitement of a peripheral nerve, which transmits its influence to certain nerve cells, and these in their turn excite certain muscles or glands into action; *and all this may take place without any sensation or consciousness on our part …*” (Darwin, 1872).

The idea that something other than the individual’s higher cognitive mechanisms could influence *behavior*, however, more or less preoccupied Darwin’s thinking. He wrote, “*when movements, associated through habit with certain states of the mind, are partially repressed by the will, the strictly involuntary muscles, as well as those which are least under the separate control of the will, are liable still to act*” (Darwin, 1872).

Darwin could not provide answers to the dilemma of peripheral control of heart rate with any certainty and concluded his thinking with a challenge to physiologists. “*From these several causes, we may conclude that the philosophy of our subject* [i.e., the expression of emotions in man and animals] *has well deserved the attention which it has already received from several excellent observers, and that it deserves still further attention, especially from any able physiologist* (Darwin, 1872).

### Attempts to Resolve Control of Heart Rate Dilemma

Prior to the publishing of Darwin’s theory of natural selection in 1859, the understanding of heart rate control had advanced significantly, particularly with the work on *vagal inhibition* by the Weber brothers (Ernst, Wilhelm, and Eduard) and the discovery of *vagal arrest* by Claude Bernard (Hoff, 1940) in the 1840’s. As a result, by the time Darwin published his theory, a new generation of physicians, physiologists and neurologists were beginning to dissect the influence of the central nervous system on the heart.

By 1850 early work had begun to reveal the workings of the central nervous system and encourage speculation on emotional behavior. In the 1860’s Russian physiologist Ivan Sechenov (1829–1905) pioneered the use of electrophysiology, demonstrating that the brain is linked to cardiac inhibition through electric currents (Stuart et al., 2014). In 1864, John Hughlings Jackson (1835–1911) proposed that the nervous system is organized in a hierarchical manner, with the cortex at the top (Jackson, 1958). Neural functions that appear first in evolution, he argued, are the ones that appear first in development and the last to disappear in disease. New neural functions are layered onto the earlier functions over the lifespan through learning. These disappear through disease, trauma and aging in a last-in-first-out manner. Jackson called his process dissolution, which he borrowed from Herbert Spencer (Berrios, 2001). Jackson showed that lesions in higher brain centers inhibited the lower centers and caused negative symptoms whereas positive symptoms were caused by emergence of the lower, subconscious centers (i.e., brain stem and visceral relays).

William James (1842–1910), among the most influential thinkers of the late 19th century, differed with Jackson’s conclusions, but like Jackson advocated for a *functional approach* to the study of emotions (James and Lange, 1922). Influenced by Darwin, James’ theory of emotions stated that emotions are the mind’s perception of physiological conditions that result from some stimulus, often internal. Danish neurologist Carl Lange (1834–1900) independently arrived at a similar theory but took the more radical position that vasomotor changes are actually *synonymous* with emotions (James and Lange, 1922). Echoing Darwin’s first and second principles of emotional expression, the James–Lange theory held that emotions arise in physiologic conditions of the body and become encoded as emotional memory by the brain.

By the dawn of the twentieth century, Darwin’s dilemma of *heart rate control* remained unresolved, with arguments falling into two distinct camps. One group of scientists (James–Lange, Pavlov) were focused on visceral control of emotional behavior and heart rate. The other group (Morgan, Wundt, Titchener, Jackson, Sherrington, Freud) favored theories that promoted cognitive control that were mostly in concert with Darwin. The field of Psychology was split between James and Lange’s idea that emotional behavior stemmed from internal signaling to the brain and Freud’s brain-based psychoanalytic theory. The Western and Russian schools of Physiology and Neurology were also split on the subject. Western researchers, influenced by Jackson and Sherrington, focused on the central nervous system and central excitation and inhibition. The Russian school, under Pavlov’s influence, focused on the autonomic nervous system and peripheral inhibition.

The work of Ivan Pavlov (1849–1936) took a viscera-centric “*subconscious*” approach to the study of emotional behavior that was increasingly at odds with Western schools of thought. Pavlov and other Russian scientists openly criticized many of the scientific methods and arguments being used and promoted in Western Psychology as being subjective, vague or confused (Pavlov, 1932; Gantt, 1960). This theoretical and methodological conflict was exacerbated by the growing political and ideological divide between Russia and the West. As a result, Russian research on autonomic conditioning was increasingly ignored and portrayed as “old school,” simplistic and obsolete (Rescorla, 1988).

Pavlov is best known for discovering his eponymous subconscious reflex conditioning mechanism, and for demonstrating the effects of that mechanism on salivation and the digestive system. Less known is Pavlov’s work on the study of conditional neurotic behavior in dogs, and the effects of Pavlovian conditioning on the autonomic nervous system and peripheral inhibition of heart rate (Pavlov et al., 1928; Gantt, 1965). Sherrington recognized that the spinal reflex is composed of integrated actions of the central nervous system involving the excitation and inhibition of many nerves, but Pavlov postulated that the origin of neurotic disturbances is principally through a collision or conflict between cortical excitation and subcortical inhibition. Pavlov, in concert with Carl Lange’s theory of emotion (see above), came to believe that subcortical reflexes and social instincts are one and the same (i.e., that the subconscious learning mechanism underlying the cardiac conditional reflex is the same learning mechanism that underlies instinctive behaviors) (Pavlov et al., 1928).

The term “conditional reflex” as used here was defined by Pavlov (Gantt, 1968). Pavlov always employed the adjective modifier “conditional” to describe the response to a stimulus, never the past participle “conditioned,” which implies being fixed. Conditional refers to the quality of the response and indicates that the response always varies with conditions (Gantt, 1968). With extreme clarity, Pavlov showed that under certain conditions the autonomic nervous system can play a dominant role in regulating emotions and inhibiting heart rate, through what he termed the “cardiac” or “social” reflex. He was the first to describe a phenomenon whereby the heart rate in dogs undergoing an emotional challenge can be significantly reduced by the physical presence of and contact with the dog’s trusted handler. Pavlov termed this phenomenon “effect of person” (Pavlov et al., 1928).

In 1927, physiologists Walter B. Cannon (1871–1945) and Philip Bard (1898–1977) dramatically altered the study of emotions with their *Thalamic theory of emotions*, which they claimed overturned the viscera-centric James–Lange theory of emotions (Cannon, 1987). The James–Lange theory proposed that emotions are generated by the physiological conditions of the body and that emotional behavior results from two-way communication between the gut and brain (Welch and Ruggiero, 2005). The new Cannon–Bard theory proposed that emotions are patterns generated in the newly discovered thalamus and that emotional behavior results *entirely* from signals transmitted from the brain to the viscera (Welch and Ruggiero, 2005). This idea and most of Cannon–Bard’s arguments were soon disputed. Nonetheless, Western science became increasingly focused on the central nervous system and higher brain function as the keys to understanding emotional disorders to the exclusion of peripheral and autonomic nervous systems.

The Russian school of physiology, under the leadership and influence of Pavlov, continued to examine conditional reflexes in the viscera and peripheral inhibitory influences on heart rate (Klimov, 1986). At this same time, Western physiologists were elucidating reflexes that evoke vagal discharge, most importantly change in blood pressure within the aorta and carotid sinus (Hoff, 1940). Neurophysiologist Sherrington described neuronal synaptic communication and demonstrated the importance of peripheral inhibition to the central nervous system. In his 1932 Nobel speech, *Inhibition as a coordinative factor*, Sherrington emphasized the problem of *peripheral* cardiac inhibition.

“*The role of inhibition in the working of the central nervous system has proved to be more and more extensive and more and more fundamental as experiment has advanced in examining it…In the working of the central nervous machinery, [peripheral] inhibition seems as ubiquitous and as frequent as is excitation itself*” (Nobelstiftelsen, 1965).

Western scientists were turning their attention to the new more exciting “cortical” learning mechanism discovered in the late 1920’s by two Polish medical students, Jerzy Konorski (1903–1973) and Stefan Miller (1903–1940) (Miller and Konorski, 1928), who argued that this mechanism was separate and distinct from Pavlovian conditioning (Miller and Konorski, 1928).

Skinner and Thorndike’s operant conditioning and Law of Effect (aforementioned) and support after two influential publications in 1948 that reinforced a growing consensus in Western science about Darwin’s puzzling over control of heart rate. First, Konorski published his monograph, *Conditioned Reflexes and Neuron Organization* (Konorski, 1948), that provided a theoretical cortical learning mechanism to control emotions through executive brain function. Second, Norbert Wiener (1894–1964) published his revolutionary book, *Cybernetics: Or Control and Communication in the Animal and the Machine* (Weiner, 1948), which reinforced John Hughlings Jackson’s argument for a hierarchical nervous system, with the cortical brain in control at the top. Weiner’s Control Theory essentially eliminated the boundary between man and machine, proposing that there is virtually no difference between the way machines and animals decide and control their actions. Both are determined by circular, causal chains, or feedback loops *within a closed system*. Behavior in humans, Weiner stated, moves from *action* to *sensing* to *comparison* with desired goal, and then to *corrective action*. The theories of Konorski and Weiner dovetailed with those of Skinner and Thorndike, and added to a postwar enthusiasm for all things cognitive, mechanistic and non-emotional. A breakthrough came in the understanding of heart rate when it was linked to respiratory rhythms by Hering in 1910 (Hering, 2013), however, the significance of this phenomenon was not clear.

By the 1940s, however, two other American behaviorists, B. F. Skinner (1904–1990) and Edward Lee Thorndike (1874–1949), were modifying the ideas of Watson and embracing Konorski’s cognitive learning mechanism. Skinner believed that Pavlovian conditioning reflex was too simplistic to explain complex human behavior. He looked for the causes of an action and its consequences, calling his cognitive learning approach *operant conditioning* (i.e., changing behavior by following a desired response with reinforcement) (Skinner, 1938). This method followed Thorndike’s *Law of Effect*, which states behavior that is reinforced tends to be repeated and behavior that is not reinforced tends to die out or be extinguished.

While many Western scientists continued to believe that Cannon and Bard had overturned James–Lange’s visceral theory of emotions, physiological evidence was accumulating that challenged many, if not all, of their arguments [e.g., that the viscera are too insensitive, uniform and slow to offer a satisfactory means of influencing emotions (Lang, 1994; Pessoa and Adolphs, 2010)]. At the same time, Pavlov’s ideas about autonomic conditioning and studies on experimental neuroses in dogs had gained a foothold in the West. They were continued and advanced through the efforts of physiologist W. Horsely Gantt (1892–1980) at his Pavlovian research lab at Johns Hopkins Medical Center. Gantt had studied with Pavlov in Russia for many years and was translating Pavlov’s work into English. In 1948, the same year Konorski and Weiner were helping build a scientific consensus around cortical learning and self-regulation, respectively, Gantt published a paper entitled *Physiological Psychology.* Sensing that the tide of argument was shifting toward the central nervous system, Gantt argued strenuously that, “*The role of the subcortical and segmental nervous system in mammals remains unsettled*” (Gantt, 1948). He argued that psychologists should maintain a holistic approach to the study of behavior by examining the physiology of the entire organism.

By 1950, the debate over whether the brain or the body controls our emotions had consolidated into an “orthodox” view that *emotional behavior* is self-controlled by higher brain regions within a closed neural system of feed-back signaling loops. Neo-Darwinism reached its pinnacle of expression in the *Modern Synthesis* of evolutionary biology by Julian Huxley (1887–1975) and others. The synthesis stated their position succinctly in three unequivocal tenets (Kutschera and Niklas, 2004): (1) Instructions for building organisms reside in genes; (2) Genes are the exclusive means by which these instructions are faithfully transmitted from one generation to the next; (3) There is no meaningful feedback from the environment or the experience of the organism to its genes.

Not all scientists, however, especially physiologists, agreed with this consensus. Horsley Gantt and his colleagues continued their experiments on peripheral inhibition of heart rate and the *effect of person* phenomenon (Gantt, 1972). Notable psychologists remained unconvinced, as well. Some were taking up Freud’s challenge to seek biological mechanisms for his subconscious theories in fields other than Psychology. A new group of researchers, calling themselves developmental psychobiologists, echoing William James, believed that psychological processes have biological or physiological correlates. These researchers criticized behaviorism for focusing on externally observable behavior and not taking into account subconscious internal influences on behavior. Influenced by the work of Tinbergen, which codified the methods for studying instinctive behavior in the natural habitat (Tinbergen, 1951), psychobiologists such as Daniel S. Lehrman (1919–1972) were beginning systematic studies of the physiology underlying instinctive behaviors of the mother and infant in the early postnatal period (Silver and Rosenblatt, 1987).

The last half of the 20th century saw major advances in the understanding of the autonomic nervous system and control of heart rate. Whereas, psychophysiological research on autonomic cardiac inhibition associated with emotional behavior was previously tied to and influenced by sympathetic activity (Duffy, 1957), new research was beginning to challenge this idea, especially new discoveries into the function of heart rate variability and respiratory sinus arrhythmia. There was increased interest in “slow breathing” techniques that were practiced in Eastern disciplines to control heart rate (Zaccaro et al., 2018). New anatomical discoveries and new phylogenetic and functional insights on the vagus in emotional behaviors gradually emerged between the 1960 and 1995 (Sokov, 1963; Lacey and Lacey, 1978). These found their synthesis in the *Polyvagal Theory* of Porges (1995, 2011).

Polyvagal Theory emphasized that humans utilize two distinct vagal systems to control heart rate in response to threat or fear; a phylogenetically older reptilian system, and a more newly evolved mammalian vagal system. “The behavioral derivative of the two branches of the vagus,” Porges wrote, “suggests a typology in which one branch of the vagus deals with unconscious reflexive functions and the other is involved in more conscious, voluntary, flexible, and often social activities” (Porges, 2011). The two neural pathways are theorized to regulate autonomic state and the expression of emotional and social behavior through a closed system of feed-back loops, with the newer mammalian vagus controlled via the cortex and the older vagus controlled via autonomic state (Porges and Kolacz, 2018; Porges and Lewis, 2018).

Importantly, Porges pointed out that strong emotion can “instantly” affect heart rate, independent of the spinal cord and the sympathetic nervous system, which in turn can affect the brain and behavior through afferent feedback signaling. In this respect, Polyvagal Theory advanced ideas first developed by Darwin and later by William James (Colzato et al., 2017). However, the theory does not fully resolve Darwin’s dilemmas. The theory, for example, does not provide a mechanism to account for learned “unconscious reflexive functions.” Nor does it provide a definition of instinctive behavior. Rather than fully resolving these questions, Polyvagal Theory provides a new expanded evolutionary framework within which to view heart rate and the role of the autonomic nervous system, and within which intriguing questions can be asked. For instance, can the “instant” direct heart rate phenomenon described by Porges be related to Pavlov’s *effect of person* phenomenon?

Pavlov’s *effect of person* phenomenon, as mentioned above, was being separately and extensively studied throughout the post-war years into the 1980’s. Horsley Gantt and his collaborators at Johns Hopkins found the effect within and between multiple species, including humans (Lynch and Gantt, 1968; Gantt et al., 1991). Gantt tried to convince his peers that the “effect of person” phenomenon could have enormous implications for the treatment of emotional disorders (Gantt, 1972). He argued the phenomenon had particular relevance to heart rate control, since his research had shown conclusively that contact with a trusted other person can have profound “instant” inhibitory effects on the spike in heart rate in the face of emotional challenge.

Gantt’s findings pointed to a radically different view of heart rate control. In matters of deep emotional challenge, Gantt’s team showed that heart rate is not solely controlled by conscious control within an individual closed system. Rather, heart rate is subject to a subconscious conditional reflex formed between an individual and a trusted other. Gantt came to believe that heart rate of the individual is subject to a subconscious calming reflex during physical contact with a trusted other. Interestingly, Darwin was familiar with the *effect of person* phenomenon. He related a story told to him by his father, a physician, about the exceptional calming effect that he himself had on a patient with heart disease. The patient complained that his pulse was habitually irregular to an extreme degree, *except when Darwin’s father entered the room*, whereupon he was calmed and his heart rate invariably became regular (Darwin, 1872).

The growing field of epigenetics is beginning to challenge some of the prevailing ideas about instincts and evolution. New insights from sociobiology are expanding understanding of complex behavior systems in animals and insects, and discovering that Darwin’s social instincts – altruism and cooperation – actually provide an advantage for the survival of some groups. While intriguing, these advances in knowledge raise as many questions as they do answers. In the past two decades, advances in computer science, technology and data analysis have made it possible to examine body-wide developmental systems and the physiological relationships between mother and infant in ways never before possible, bringing us closer to the ability to understand how peripheral control of heart rate relates to innate behavior or to the mechanisms involved.

## Part 3: Proposed Resolution of Darwin’s Dilemmas

Whether the aforementioned theorists agreed with Darwin’s theory of emotion or not, most sought to understand emotional behavior and provide a biological mechanism to account for it, especially during the perinatal period. Most tried to account for so-called phenotypical variation that occurs in infant and early childhood behavior. Why does one infant or child display pro-social behavior and another anti-social behavior? Is such phenotypical behavior fixed, or can it change? If phenotypical behavior can be changed after birth, what biological mechanism(s) account for the change?

We now critically review three currently influential theories on emotional behavior and review two theories that offer a new resolution to Darwin’s instinct and control of heart rate dilemmas.

### Attachment Theory

It was in response to the questions outlined above that Bowlby developed his attachment construct in response to government and social service organizations’ struggle to find a practical solution to antisocial behaviors displayed by large numbers of children who had been separated from their families during WWII. The construct was designed to describe a system of behavior that could be acted upon in order to change antisocial to social behavior. As pointed out above, Bowlby believed that he had created a new way to describe the behavior of infants and children that was in line with biological science at the time. As Mary Ainsworth put it,

*The great strength of attachment theory in guiding research is that it focuses on a basic system of behavior, the attachment behavioral system, that is biologically rooted and thus species-characteristic. This implies a search for basic processes of functioning that are universal in human nature, despite differences attributable to genetic constitution, cultural influences, and individual experience* (Ainsworth, 1989).

Bowlby, however, was not a basic scientist. He relied upon scientific theories and evidence of others at the time to provide the mechanism underlying attachment behaviors. The practical problem of finding a way to identify and measure the attachment behavioral system fell to Ainsworth (1969), a developmental psychologist, who created a system of identification. Ainsworth originally described three attachment types, *Secure, Insecure-Avoidant* and *Insure-resistant*, to which *Insecure-disorganized* was later added by others (Benoit, 2004) (see Table 1).

**Table 1:** Attachment styles and their origin.

	Secure	Insecure		
		
		Avoidant	Resistant	Disorganized
Caregiver’s Style	Loving	Rejecting	Inconsistent	Atypical
	↓	↓	↓	↓
Offspring’s Coping Strategy	Organized	Organized	Organized	Disorganized


Today, Bowlby’s attachment construct and Ainsworth’s system for coding attachment behavior are the accepted method by which the majority of research psychologists categorize the behavior of infants and children. Bowlby’s attachment construct describes an individual system of behavior separate from the mother. It states that the mother/child relationship does not have to be reciprocal and that the mother should serve as a secure base from which the child moves off according to the child’s priorities (Bowlby, 1969). However, the attachment field has struggled to associate the categories with physiological correlates in order to provide a simple explanation of the differences of the four attachment behaviors. Mechanisms, when they are proposed are apt to be behavioral (Moretti et al., 2015), or cognitive-based and complicated (Laurita et al., 2017).

While there have been notable recent attempts to revise attachment theory to align with growing evidence that challenges it (Schore, 2005; Lickliter, 2008; Feldman, 2017), the attachment construct remains firmly grounded in the assumptions that emotional behavior is controlled within the individual organism through self-regulation and subject to cortical control. Brain imaging studies are seeking to associate brain function with attachment style (Pratt et al., 2018), or by dropping the heralded four types of attachment and simply dividing subjects between secure and insecure attachment (Saunders et al., 2015). Interestingly, researchers looking at mechanisms in the neonatal period have begun focusing on the association between cardiac function and attachment (Sancho-Rossignol et al., 2018). Nevertheless, the attachment construct has failed to deliver a practical tool for clinicians (Flores et al., 2018; Valikhani et al., 2018). Attachment coding methods require substantial training, are time-consuming and costly to administer, and are not scalable as a clinical tool.

Whether one agrees or disagrees with this critical assessment of attachment theory, one must conclude that the attachment construct has not been the breakthrough on instinct that Bowlby heralded it would be in 1960. Nor has Bowlby’s theory fulfilled his promise to answer Freud’s call for a mechanism underlying emotional behavior. Importantly, as Hofer and others noted, attachment as a unique motivational system has not generated a simple testable hypothesis (Hofer, 2006). In any case, Bowlby’s reliance on analytical biology, gene theory and control theory has produced an increasingly complex and opaque view of the basic principles that govern mother-infant emotional behavior.

**FIGURE 4:**
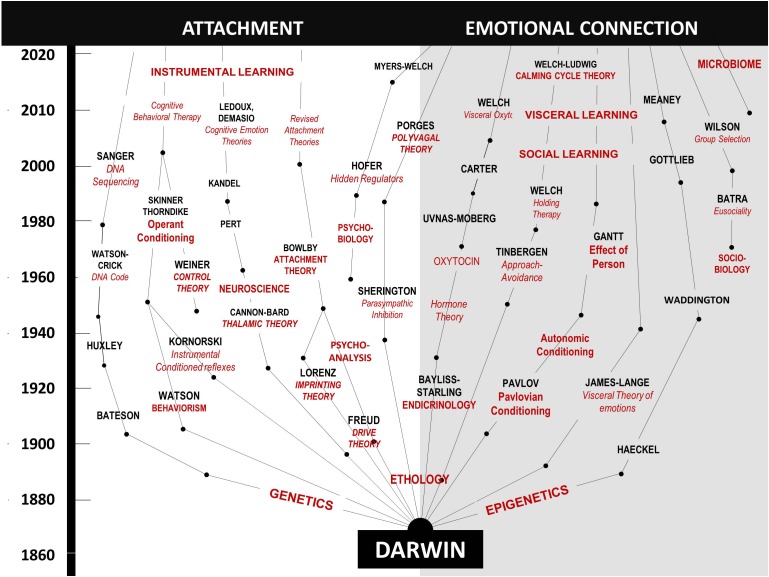
A diagram showing the contrasting development of theories related to the dilemmas of instincts and control of heart rate since Darwin. On the left, are those theories leading to the behavioral construct of attachment. On the right are theories leading to the behavioral construct of emotional connection. The two sides highlight scientific arguments and theoretical conflicts in multiple fields, as outlined in Table 2.

In Figure 4, on the left side, we trace the theoretical path that led from Darwin to the attachment construct. Note that this path is dominated by *psychology and research focused on the central nervous system*, leading to the attachment construct. In contrast, on the right side, we trace the theoretical path to the emotional connection construct and calming cycle theory, a path dominated by *physiology and research focused on the autonomic nervous system* (see Table 2).

**Table 2:** Comparison of theories supporting attachment and emotional connection by category.

Area of Interest	Attachment	Emotional Connection
Behavior	Psychiatry, neuroscience, ethology	Physiology, endocrinology, sociobiology, microbiology, ethology
	*Freud, Bowlby*	*Pavlov, Porges, Welch*

Cell signaling	Central nervous system	Autonomic nervous system
	*Sechenov, Sherrington, Kandel*	*Sherrington, Porges, Welch*

Learning mechanism	Operant conditioning	Pavlovian conditioning
	*Konorski, Skinner, Thorndike*	*Pavlov, Gantt, Welch-Ludwig*

Evolution	Genetic	Epigenetic
	*Bateson, Sanger*	*Haeckel, Gottlieb, Meaney*
	
	Natural selection	Group selection
	*Darwin, Smith*	*Batra, Sober and Wilson*

Control of Emotions	Self-regulation, Cognitive	Co-regulation, Visceral
	*Canon-Bard, Skinner, Thorndike, Weiner, Damasio, Ledoux*	*James–Lange, Pavlov, Gantt, Welch*

Instincts	Genetic, Inherited	Environmentally Learned, epigenetics
	*Freud, Bowlby*	*Waddington, Tinbergen, Gottlieb, Meaney*

Control of Heart Rate	Cortical – central nervous system	Sub-cortical – parasympathetic nervous system
	*Sechenov, Sherrington, Damasio, Ledoux*	*Morgan, Pavlov, Porges*


### Evolutionary Game Theory

Evolutionary game theory provides an explanation for Darwin’s social instincts, by identifying how issues of cooperation and defection can profoundly affect strategies of survival. This epigenetic process played out over multiple generations is theorized to favor kin relationship and, therefore, introduces the idea of inclusive fitness, which includes an individual’s offspring as well as any offspring equivalents found in kin. In this way, the genetic makeup of the species is determined by repeated games of competition for survival, which happen to include the traits of altruism and cooperation usually found within kinship.

We see several limitations of evolutionary game theory and indirect reciprocity regarding the theoretical problems posed by Darwin’s writings on emotional behavior. First, the application of equations from mathematics and physics to problems in biology *per se* is subject to limitations of reductionist theories in general. Gyorgy Szabo summarizes this limitation at the end of his impressive and exhaustive review of evolutionary game theory (Szabo and Fath, 2007), “Unfortunately, systematic investigations are strongly limited by the wide variety of microscopic rules and the large number of parameters.” Reducing biological processes to mathematics and equations is an activity no doubt valid for some applications. But, at a certain point, the process fails to have practical relevance to infants.

In the context of our review, evolutionary game theory fails to solve the most fundamental question that baffled Darwin regarding perinatal social instincts. Where do instincts come from and how are they preserved evolutionarily? Aside from considerations of evolution, these are questions that have special current relevance and urgency in the field of neonatology, where prematurely born infants suffer disproportionately from emotional and behavioral disorders.

Evolutionary game theory purports to consider behavioral and biological mechanisms, but to our knowledge the field is mostly concerned with how populations evolve at the genetic, individual and group levels, by investigating the strategic mechanisms involved in various competitions for resources between organisms. The theory is less concerned with how to intervene in a particular “competition” in order to change the outcome. On this matter, the theory is agnostic.

We in no way mean to demean or slight evolutionary game theory or indirect reciprocity. There are many fields and applications that have benefited and will continue to benefit from the theories. Even the fields of human behavior and emotions have been enriched by the theories. However, all the theories on emotions from Darwin to present, including evolutionary game theory, have failed to produce a simple testable hypothesis for a biological mechanism that can account for the origin of social instincts in the perinatal period, or to provide an intervention that can overcome maladaptive behavior when it arises.

### Polyvagal Theory

Polyvagal theory, originally conceptualized by Stephen Porges, has increased understanding of vagal inhibition of heart rate and advanced new ways of assessing the role of the autonomic nervous system in emotional behavior, and provided a system to measure autonomic health in the individual (Kirby et al., 2017; Mulkey and du Plessis, 2019).

Importantly, Porges’ theory is supported by recent clinical innovations that take advantage of a *vagal mechanism* that utilizes an *inflammatory reflex* (Tracey, 2002), building on Pavlov’s research on the cardiac reflex. The growing field of vagal nerve stimulation is drawing attention to the powerful influences of the autonomic nervous system on the control of inflammation and hormone release (Reyes-Lagos et al., 2018), including oxytocin (Kenkel et al., 2014).

In our effort to fully explain instinctive behavior and control of heart rate, we are guided by ethologist Niko Tinbergen. To understand any behavior fully, Tinbergen proposed that we need to answer four questions (Tinbergen, 1963):

(1) What is the *evolution or phylogeny* of the behavior, i.e., how might it have arisen?
(2) What is the *adaptive value* of the behavior, i.e., how does it impact the animal’s chances of survival and reproduction?
(3) What is the *causation or mechanism* of the behavior, i.e., what stimuli elicit the response, and how is it modified by learning?
(4) What is the *development or ontogeny* of the behavior, i.e., how does it change based on genetic and epigenetic factors?

Polyvagal theory answers question 1, how the mammalian polyvagal system evolved to modulate emotional behavior and control of heart rate (Porges, 1995), and question 2, what the adaptive value of the vagal system is (Porges, 2011).

As Porges points out, modulation of heart rate and the *social engagement system* matures before birth during the third trimester of gestation, when the myelination of the fetal vagal system matures.

When vagal tone, through myelinated vagal pathways, to the [cardiac] pacemaker is high, the vagus acts as a restraint, or brake, limiting the rate at which the heart can beat and functionally calming the individual. When vagal tone to the pacemaker is low, there is little or no inhibition of the pacemaker, and the heart rate increases. The vagal brake construct may be used to describe functional modulation of heart rate by myelinated vagal efferent pathways (Porges, 2011).

Vagal tone can be used to determine health. Heart rate and the amplitude of respiratory sinus arrhythmia (RSA) are subject to direct vagal mechanisms. However, there are situations in which the measures appear to reflect independent sources of neural control. This paradox in the data remains the subject of debate among physiologists. The paradox is described thus: Increased vagal tone can produce neurogenic bradycardia. Decreased vagal tone produces suppression of RSA. However, bradycardia can occur during periods of suppressed RSA.

This vagal paradox presented by the physiologists’ data had broad clinical significance. If vagal tone is a positive indicator of fetal or neonatal health when monitored with RSA, then why is vagal tone a negative indicator of health when it includes bradycardia? This paradox provided the stimulus for the development of Porges/polyvagal theory, which identifies the relationship between visceral experiences and parasympathetic vagal control of the heart (Porges, 1995).

Porges lists five explanations for the vagal paradox, all based upon the assumption that there is a single common source of cardiac vagal tone:

(1) RSA and average heart rate (during sympathetic blockade) reflect different dimensions of vagal activity.
(2) RSA is being confounded by respiratory frequency and tidal volume.
(3) Variation in quantification methods may contribute to the divergence between RSA and heart rate.
(4) RSA does not reliably measure parasympathetic tone because it decreases with baroreflex stimulation.
(5) Average heart rate is influenced by a complex and dynamic interaction between sympathetic and vagal systems, making it difficult to extract a vagal tone dimension.

Alternative explanations proposed by polyvagal theory, also based upon a single source, suggest that cardiac vagal tone may vary from person to person and according to varying conditions. However, a convincing explanation for the vagal paradox has not emerged based on the assumption that cardiac vagal tone derives from a single source.

### Calming Cycle Theory

Having reviewed efforts to resolve Darwin’s dilemmas of instincts and control of heart rate with the assumption that emotional behavior is self-regulated in a top-down fashion within a closed system of feed-back loops, we will now propose a resolution based upon a new set of assumptions. This work came out of Welch’s aforementioned clinical insights in the 1970s through the 1990’s, and summarizes concepts that emerged from basic and clinical research in the BrainGut Initiative and the Nurture Science Program at Columbia University Medical Center over the past twenty years.

Calming cycle theory is first and foremost a *theory of change*. It provides the answers to Tinbergen’s question 3 by explaining the causation and mechanism of so-called instinctive behavior in the perinatal period and how the vagal system works mechanistically on heart rate to produce instinctive behaviors (Welch, 2016; Welch and Ludwig, 2017a). Calming cycle theory also provides the answer to Tinbergen’s question 4 by explaining the development or ontogeny of instinctive behavior and how phenotypical maladaptive behaviors can be changed, based on facilitated interactions between mother and infant (Welch and Ludwig, 2017b).

Calming cycle theory advances understanding of the vagal system in humans. For instance, the theory proposes a novel explanation for the *vagal paradox* by proposing a second and separate source of regulation of infant cardiac vagal tone; *the mother*. According to calming cycle theory, vagal tone and heart rate are *co-modulated* within an *open co-regulatory feedback system* between mother and fetus/infant (Welch, 2016). In a normal and healthy gestation, an autonomic reflex is created during gestation that co-regulates, among other physiological processes, heart rate. Autonomic co-conditioning continues after birth, producing a co-regulatory parasympathetic cardiac calming reflex. Importantly, perinatal physiological co-regulation is conditional upon emotional connection (see below), which ensures that mother and infant seek proximity to one another after birth.

Our proposed solution to Darwin’s heart rate dilemma is consistent with the large amount of evidence accumulated by Pavlov and Gantt documenting the cardiac or social reflex and the *effect of person*, as cited above. Animal research has shown that normal mother–infant interactions are associated with autonomic regulation. For instance, physiological co-regulation has long been viewed as inherent to optimal mother–infant interactions (Winberg, 2005). Other researchers, such as Uvnas-Moberg et al. (1987) and Hofer (2006), found that stimuli emanating from the infant also serve as regulators of maternal physiology. Prior vagal research may also support this model. For instance, Porges et al. (1996) demonstrated that infants with difficulty decreasing vagal tone (the vagal brake) during social/attention tasks at 9 months had more behavioral problems at 3 years of age.

In healthy relationships, proximity of mother and infant activates a parasympathetic calming reflex that results in higher vagal tone (Field and Diego, 2008). These proximity-seeking behaviors are what have traditionally been associated with instinctive or innate behaviors. We hypothesize these mother/infant proximity-seeking behaviors are the result of Pavlovian co-conditioning of the cardiac calming reflex that occurs in utero. Accordingly, instincts are not inherited, but are environmentally shaped in utero and continue to be environmentally shaped postnatally.

According to calming cycle theory, the ability to suppress vagal tone to attend to one another (social/attention tasks) is conditional on emotional connection or the lack of connection. Lack of emotional connection (i.e., through physical or emotional separation) produces various *self-regulatory* mechanisms of mother and infant and devolves into dysregulation. According to calming cycle theory, the vagal paradox data reflects a break in co-regulation, which produces a *dysregulated autonomic state*. Such a dysregulated state can be resolved through co-regulating contact with the mother. This hypothesis that can be easily tested, as shown below.

According to calming cycle theory, the mechanism underlying instinctive or innate behavior can be summarized with these four postulates:

Postulate 1. Subcortical Pavlovian co-conditioning of the autonomic nervous system of mother and infant during gestation leads to a perinatal calming reflex and to emotional connection.
Postulate 2. Autonomic calming reflexes between mother and infant are activated upon contact and result in positive socio-emotional behaviors (Welch and Ludwig, 2017a).
Postulate 3. The autonomic calming reflex can be broken or interrupted by physical and/or emotional separations between mother and infant during the perinatal period, producing emotional disconnection and negative socio-emotional behavior.
Postulate 4. Disrupted or maladaptive autonomic behavioral reflexes can be counter co-conditioned through regular and repeated mother–infant calming interactions, or calming cycles.

We do not mean to say that there are no cortical influences on these processes. Sensory processing by the cortex, amygdala, and many brain circuits are inherently involved in certain aspects of behavioral regulation. As Gantt showed, however, the cognitive and autonomic systems function separately (Gantt, 1953). We posit that anomalies in behavioral (and vagal) characteristics seen during the perinatal period, including those viewed as pathological and often attributed to disorders of cortical/cognitive mechanisms, are in fact disruptions of subconscious, autonomic mechanisms.

**FIGURE 5:**
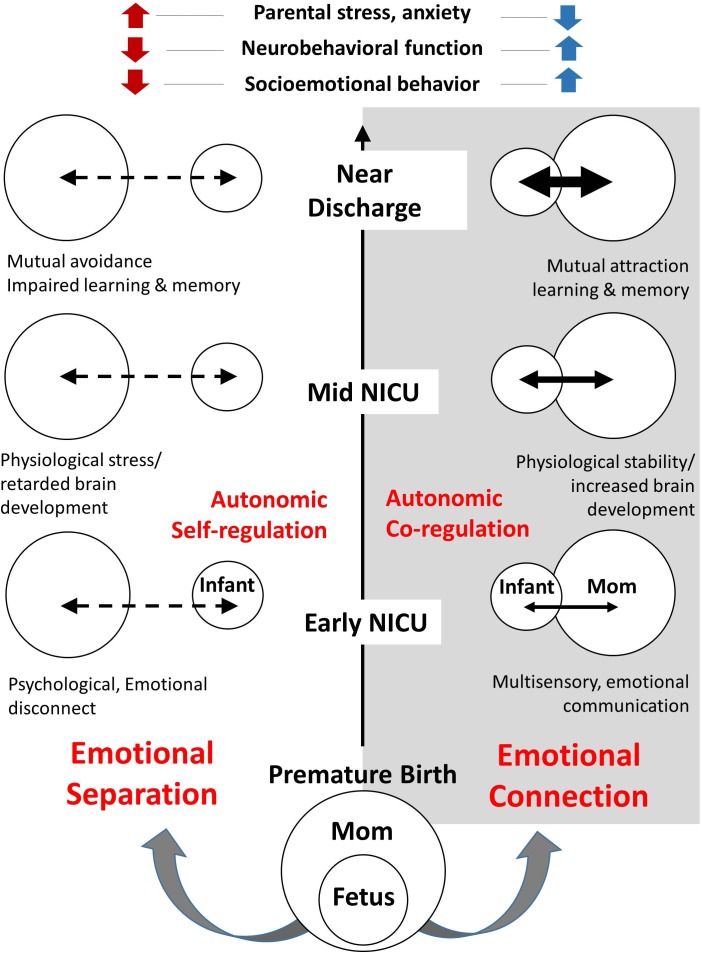
Schema showing the positive effects of facilitating and maintaining emotional connection between mother and infant in the NICU (SC = standard care; FNI = standard care plus Family Nurture Intervention).

**FIGURE 6:**
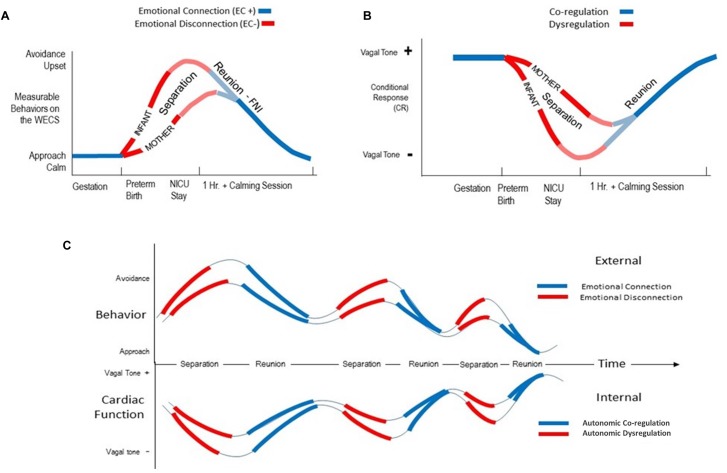
Hypothesized relationship between perinatal behavior and cardiac function. Panel **A** depicts dyadic behavior over time in the NICU. Traumatic separation results in a break in emotional connection. Calming session restores dyadic calming. Panel **B** depicts the hypothesized autonomic cardiac function over time in NICU. Panel **C** depicts behavior and cardiac function together. The hypothesized calming cycle over time conditions the positive cardiac reflex and leads to emotional connection.

Recent evidence supports our proposed calming cycle theory. Randomized controlled trials of Family Nurture Intervention (FNI) in the neonatal intensive care unit (NICU) tested a theory of change based on calming cycle and emotional connection theories (see Figure 5). In the trials, infants born at 26–34 weeks postmenstrual age were randomly assigned to one of two groups; standard care alone or standard care plus FNI (Welch and Myers, 2016). FNI is narrowly focused on re-establishing an emotional connection between the mother and infant after traumatic separation caused by premature birth and NICU isolation, while the infant is still in the hospital. This strategy hypothesizes that repeated facilitated calming sessions between mother and infant in the NICU aimed at emotional connection will co-condition an adaptive autonomic reflex in both the mother and infant in response to contact with one another (see Figure 6).

The first FNI-NICU trial and calming cycle study (clinicaltrials.gov, NCT01439269) subsequent ongoing multi-site trial (clinicaltrials.gov, NCT02710474) and an effectiveness trial (clinicaltrials.gov, NCT02710474) are important for two main reasons. First, they have provided evidence that supports calming cycle and emotional connection theories, i.e., significant short and long-term outcomes in infant/child and mother across multiple domains (Welch et al., 2014b, 2015, 2016; Hane et al., 2015). Second, they are providing the opportunity to test our theories about instinctive behavior and control of heart rate in a human population at high risk for life-long socioemotional disorders. These hospital-based trials are arguably the most rigorous and comprehensive studies of any intervention between mother and infants in the NICU to date. Analyses of data through 5-years follow-up in the first trial are ongoing.

**FIGURE 7:**
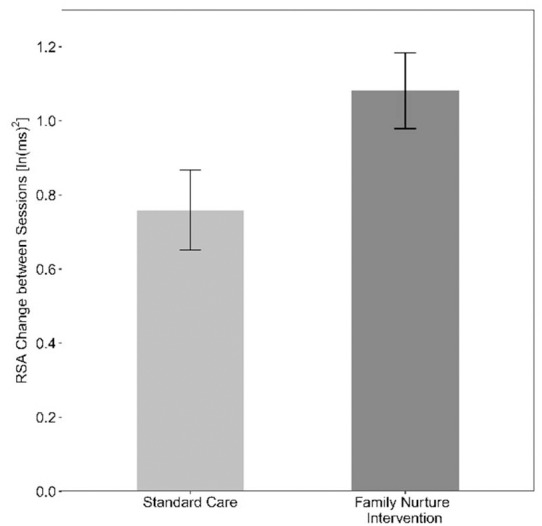
As the infants approached term, increases in RSA were significantly greater in infants receiving FNI compared to the SC group (*p* = 0.031; Cohen’s *d* = 0.38; error bars represent standard error).

In collaboration with Porges et al. (2018), we tested the hypothesis that facilitating repeated calming sessions will lead to improved autonomic function in the infant. Analyses of the FNI-NICU trial data showed that FNI significantly enhances infant autonomic regulation after 2 to 6 weeks of FNI in the hospital (Porges et al., 2018), compared to infants receiving standard care. In the study, electrocardiograms (ECG) were collected for approximately 1 h during sleep at two time points, approximately 35 and 41 weeks postmenstrual age. Heart rate and RSA were quantified from the ECG. Across the two time points, the FNI group exhibited greater increases in RSA and slope between RSA and heart rate, a measure of vagal efficiency. FNI infants demonstrated enhanced autonomic regulation consistent with greater maturation of cardiac function (Figure 7).

These cardiac findings are significant for several reasons. Prematurely born infants have maturational delays in several neurobehavioral systems, which negatively impact long term emotional, behavioral and developmental outcomes (Baron et al., 2012). The fact that a significant change in infant autonomic regulation occurred following a relatively small amount of intervention, an average of 6 h of facilitated calming sessions over an average 7 weeks, is remarkable. It suggests that FNI is acting directly upon a powerful biological mechanism.

In another analysis of FNI-NICU data, we tested the hypothesis that the emotional connection between mother and infant in the NICU intervention would lead to improved socioemotional behaviors between mother and infant at 4 months using the still face experiment, first developed by Tronick et al. (1978). Beebe et al. (2018), performed microanalysis on FNI trial data and provided evidence that supports the hypothesis. FNI dyads displayed more attraction behaviors and more sensitivity, compared with controls. In addition, the data supports the proposed mother-infant subcortical co-regulatory mechanism. The study found that at 4 months, importantly, the millisecond reaction-times of eye contact between mother and infant strongly suggest that the behaviors are not cortical in origin. The timing of the reactions indicate that they may be the result of LeDoux’s faster sub-cortical signaling network (LeDoux and Brown, 2017), as predicted by calming cycle theory (Welch, 2016; Welch and Ludwig, 2017a,b), as well as by Polyvagal Theory (Porges, 1995; Porges et al., 1996).

Apart from clinical trials, we have conducted basic research based on calming cycle theory. The translational work of the BrainGut Initiative and the Nurture Science Program has elucidated the function of oxytocin in the gut, which has been consistent with the proposed neuroendocrine co-conditioning mechanism. Oxytocin has long been associated with perinatal physiology of both mother and infant (Welch and Ludwig, 2017a). We were the first to demonstrate the presence and functions of the oxytocin receptor in the gut epithelium and in the neurons of the enteric nervous system, in both of which the oxytocin receptor is developmentally regulated (Welch et al., 2014a). Welch also showed the efficacy of treating peripheral inflammation with “peptides of nurture,” a combination of oxytocin and secretin (Welch et al., 2010) and showed the downstream effects of oxytocin/oxytocin receptor signaling in gut and brainstem (Klein et al., 2017, 2018). In addition to its many other effects, oxytocin has been shown to enhance approach behaviors (Spengler et al., 2017). Also, gastric neurohormones have been shown to affect vagal function (Chu et al., 2013; de Lartigue, 2014). Taken together with the literature, this body of work points to a complex mechanism by which mother-infant calming may be co-regulated by visceral/autonomic function, neurohormones in dyadic interactions.

### Emotional Connection Theory

We have proposed a new behavioral construct – *Emotional Connection* – that is distinct from other currently accepted constructs. The construct describes emotional behavior between mothers and infants/children (Welch, 2016; Welch and Ludwig, 2017a,b). We have demonstrated that the emotional connection construct is quickly measured and immediately actionable (Hane et al., 2019). Analogous to the force between two magnets, emotional connection describes the force that attracts two individuals to one another and keeps them together. A polar force, absence of emotional connection, holds the two apart.

Emotional connection is distinct among current behavioral constructs in that it is a two-way phenomenon in which reciprocity is an absolute requisite. Emotional connection is also behaviorally and physiologically distinct in that it describes an *open feedback system* of behaviors and physiology between mother and infant. The eco-evo-devo examination of symbiosis (see Figure 2 above) supports our view that symbiotic emotional connection and autonomic co-regulation between mother and infant determines phenotype. By changing a mother/infant relationship from weak emotional connection to strong emotional connection, the phenotype can be changed through repeated calming sessions, as described above.

The emotional connection construct has long been is supported by ethologist’s observations of approach and avoidance behaviors in animals (Tinbergen, 1951). Schneirla and Rosenblatt (1961) further researched and reported on the phenomenon. It was Tinbergen’s work on approach-avoidance that inspired Welch’s clinical insights. The *Welch Emotional Connection Screen (WECS)* was developed to assess emotional connection (Hane et al., 2019). Behaviors displayed during close physical face-to-face proximity, i.e., eye contact or gaze aversion, physical attraction or avoidance, vocal cooing and soothing or distress, reciprocal responsiveness, can be used to determine mutual or impaired emotional connection between a mother and infant.

The emotional connection construct provides a new and simple way to view the mother/infant–child relationship and understand the mechanisms that underlie it. It has practical relevance to neonatologists, pediatricians, therapists and others in the healthcare field. Together with calming cycle theory, the emotional connection construct is actionable in that it points to ways to improve outcomes for infants, children and families. The calming cycle interventions provide an effective parenting tool for families to promote adaptive socioemotional development and optimal behavior throughout childhood within the family structure.

For researchers, the advantage of the emotional connection construct is that emotional behaviors assessed on the WECS are associated with measureable autonomic states and cardiac function that are directly associated with autonomic state (see Figure 6) (Hane et al., 2019). In this sense, the construct provides a clear window to the physiology underlying emotional behavior that is simple and measureable.

Prior to the ability to study fetal growth in prematurely born infants Darwin and many of his followers concluded that instinctive behavior must be genetic and inherited. However, evidence has been accumulating for the last century that the intrauterine environment profoundly influences fetal learning. As early as 1907, physician and surgeon Byron Robinson showed that fetal learning does not necessitate adult-like brain organization or even cortex (Robinson, 1907), a fact more recently elucidated by our collaborator, Michael D. Gershon, in his book *The Second Brain* (Gershon, 1999).

For instance, it has been proposed that the fetus encodes memory via subcortical networks and a mechanism that controls reflex-type reactions and the early emotional behavior of the infant (Dirix et al., 2009), and that fetal memory may have adaptive value (Hepper, 1996). Chemosensory stimuli occurring during gestation can promote the acquisition of long-term memories, which can affect behavior in the adult. These findings suggest that some likes and dislikes (e.g., a preference for licorice) expressed in adulthood can result from exposure (conditioning) in utero (Gruest et al., 2004). Finally, recent evidence suggests that innate anxiety and learned fear are both subject to modulation through abdominal vagal afferents (Klarer et al., 2014), adding further weight to theories emphasizing an important role of afferent visceral signaling in the regulation of emotional behavior.

Using data from the FNI trial, the Welch Emotional Connection Screen (WECS) was validated by Hane et al. (2019). In this study, WECS maternal scores were positively associated with maternal sensitivity and quality of vocal contact at 36 weeks (caregiving) and maternal positivity at 4 months (face-to-face). WECS infant scores of face-to-face interaction videos at 4 months positively correlated with infant social engagement and maternal positivity, when compared to scores obtained with observed behavior tracked in real time with frame-by-frame analysis. Coding the same videos with a separate set of blinded coders, infants from dyads assessed not emotionally connected displayed autonomic dysregulation and less approach-seeking behavior toward mother during interactive/play sessions of the still-face paradigm, when compared to dyads assessed emotionally connected. In a study of 6-month old full term infants, emotional connection assessed by the WECS predicted 3-year old behavior as measured on the Child Behavior Checklist (Frosch et al., 2019). Taken together, these data provide validation of the emotional connection construct and support the theoretical foundation of the WECS as a valid instrument for assessing emotional connection in preterm mother–infant dyads. The results also strongly suggest that emotional connection is a behavioral mirror of the dyad’s co-regulatory autonomic response to contact with one another (Figure 5).

The WECS assessment tool is the first instrument to correlate behaviors with the internal autonomic state. Importantly, in the Hane et al. (2019) study only some of the behaviors coded by the Ainsworth coding system, such as attraction and sensitivity, mapped onto behaviors coded by the WECS. The majority of the Ainsworth scales measured behaviors associated with higher order/cortical function. As aforementioned, Gantt showed that cortical and autonomic behaviors are distinct and separate (Gantt, 1953; Dykman and Gantt, 1997).

Together, the FNI-NICU trial data support the emotional connection and calming cycle theories. They also support a new theory of change of instinctive behavior in the NICU to optimize postnatal development of autonomic regulation and neurobehavioral outcomes in preterm infants.

## Conclusion

Although research continues to unwind the mysteries surrounding perinatal mother/infant emotional behavior, most theories on emotional behavior are still built upon the very same assumptions Darwin made in his theories of natural selection and emotions. Arguably, these assumptions have permeated Western religion and society for millennia and undergird much of today’s society, schools, scientific research agenda and popular culture. This has resulted in a resistance to or inability to reconsider the belief systems that underlie today’s science. As William Schoenfeld aptly quoted in his presidential address at the 12th annual meeting of the Pavlovian Society in 1972, *Problems of Modern Behavior Theory* (Cornfold, 1931), “If we look beneath the surface of philosophical discussion, we find that its course is largely governed by assumptions that are seldom, or never, mentioned.”

The new theories presented at the end of this review stem from a theoretical path quite different from the one that leads to the current thinking and practice in behavioral science today. One might conclude that the divergence in paths outlined in Figure 4 stems from a differential emphasis on brain and body. But, this is a false dichotomy. The real dichotomy is between *I* and *we*.

To understand social instincts from the new perspective we present here, one must change several wide-spread assumptions about the origin, development *and control* of perinatal emotional behavior; from a self-regulatory to a co-regulatory system, and from a top-down to a bottom-up perspective. This new perspective engenders practicable and scalable interventions that increase mother-infant emotional connection and autonomic co-regulation, in order to overcome a devastating and fast-growing burden of emotional, behavioral and developmental disorders that threaten the educational and care systems underpinning human society.

Emotional connection and calming cycle theories are based on new assumptions that cast the emotional behaviors between mother and infant in new light. Emotional connection theory assumes that the mother/infant emotional relationship is measureable in a way that is predictive of future health and adaptability. Calming cycle theory assumes that the relationship that emerges between mother and infant in the perinatal period is not fixed, and that maladaptive behaviors can be changed to adaptive. The significant theoretical advance of these theories is that both theories generate simple testable hypotheses.

In closing, with Schoenfeld’s admonition in mind, we disclose a final assumption that contrasts with Darwin’s. We assume that the social instincts of *sympathy, empathy, altruism and co-operation*, rather than being traits that will degrade and doom our species, as Darwin feared, are the ones that will actually save it.

## Author Contributions

RL contributed to theory, literature research and writing. MW contributed to theoretical concepts, writing and editing.

## Conflict of Interest Statement

The authors declare that the research was conducted in the absence of any commercial or financial relationships that could be construed as a potential conflict of interest.
